# Quinoa (*Chenopodium quinoa* Willd.): An Overview of the Potentials of the “Golden Grain” and Socio-Economic and Environmental Aspects of Its Cultivation and Marketization

**DOI:** 10.3390/foods9020216

**Published:** 2020-02-19

**Authors:** Viktória Angeli, Pedro Miguel Silva, Danilo Crispim Massuela, Muhammad Waleed Khan, Alicia Hamar, Forough Khajehei, Simone Graeff-Hönninger, Cinzia Piatti

**Affiliations:** 1 Department of Historical and Geographic Sciences and the Ancient World (DiSSGeA), University of Padova, 35141 Padova, Italy; viktooria94@gmail.com; 2 Centre of Biological Engineering, University of Minho, Campus de Gualtar, 4710-057 Braga, Portugal; 3 International Iberian Nanotechnology Laboratory, Av. Mestre José Veiga s/n, 4715-330 Braga, Portugal; 4 Institute of Crop Science, University of Hohenheim, 70599 Stuttgart, Germany; danilo.crispimmassuela@uni-hohenheim.de (D.C.M.); f.khajehei@uni-hohenheim.de (F.K.); simone.graeff@uni-hohenheim.de (S.G.-H.); 5 Faculty of Agricultural Sciences, University Hohenheim, 70599 Stuttgart, Germany; m.waleed.khan@hotmail.com; 6 Faculty of Natural Sciences, University of Hohenheim, 70599 Stuttgart, Germany; aliciahr@alumni.stanford.edu; 7 Institute of Social Sciences in Agriculture, University of Hohenheim, 70599 Stuttgart, Germany; cinzia.piatti@uni-hohenheim.de

**Keywords:** quinoa, *Chenopodium quinoa* Willd., quinoa market, producer and consumer welfare, sustainability, functional food, nutrition, post-harvest processing, side stream processing

## Abstract

Quinoa (*Chenopodium quinoa* Willd.) is native to the Andean region and has attracted a global growing interest due its unique nutritional value. The protein content of quinoa grains is higher than other cereals while it has better distribution of essential amino acids. It can be used as an alternative to milk proteins. Additionally, quinoa contains a high amount of essential fatty acids, minerals, vitamins, dietary fibers, and carbohydrates with beneficial hypoglycemic effects while being gluten-free. Furthermore, the quinoa plant is resistant to cold, salt, and drought, which leaves no doubt as to why it has been called the “golden grain”. On that account, production of quinoa and its products followed an increasing trend that gained attraction in 2013, as it was proclaimed to be the international year of quinoa. In this respect, this review provides an overview of the published results regarding the nutritional and biological properties of quinoa that have been cultivated in different parts of the world during the last two decades. This review sheds light on how traditional quinoa processing and products evolved and are being adopted into novel food processing and modern food products, as well as noting the potential of side stream processing of quinoa by-products in various industrial sectors. Furthermore, this review moves beyond the technological aspects of quinoa production by addressing the socio-economic and environmental challenges of its production, consumption, and marketizations to reflect a holistic view of promoting the production and consumption of quinoa.

## 1. Introduction

Quinoa (*Chenopodium quinoa* Willd.) is a herbaceous plant, more specifically a tetraploid and halophytic crop. Quinoa is part of the Dicotyledoneae class, *Chenopodiaceae* family, *Chenopodium* genus, and quinoa species [1]. Cultivation of quinoa is indigenous to the South American Andes region, dating from 5000 BC to 3000 BC [2,3]. Throughout the history of the Inca civilization, quinoa was considered to be a sacred food [3]. The role of quinoa, however, changed during the Spanish colonial period. Given its cultural and religious links, Spaniards saw it as ‘non-Christian’, and therefore looked to replace it with other cereals [4,5,6]. Thus, quinoa production, use, and consumption started to decrease in urban settings, but quinoa cultivation in the communal lands (‘aynokas’) was preserved. This led to the development of different varieties of quinoa, depending on the localization of these ‘aynokas’. These different varieties offer different nutritional profiles as well as different visual aspects [3,5,7].

Over the past decades, quinoa production started to steadily increase, and by 2013, which was the international year of quinoa, production and consumption of quinoa increased exponentially [8]. It has gained worldwide growing attention, not only due to its nutritional and functional properties but also because of its ability to be cultivated under adverse climate conditions. Quinoa plants show tolerance to frost, salinity, drought, and have the ability to grow on marginal soils. These characteristics are therefore very relevant for areas prone to food insecurity. The recent interest in this crop increased in the last decades due to its outstanding plasticity to adapt to different environmental conditions. However, response mechanisms and adaptation strategies such as morphological (phenotypic flexibility), physiological (growth regulation), and molecular (activating stress proteins) ones developed by quinoa lead to an alteration on plant composition [9], and not to mention alterations of natural and social environments [10].

With an increasing demand and production in other countries, quinoa’s native regions which mostly rely on quinoa production for nutrition and economic survival, might suffer negative consequences due to this interest. There are also ethical and moral implications that need to be considered regarding quinoa, and they will be approached in more detail later on. Nevertheless, the production of quinoa can go to great lengths in improving food security due to the key aspects related to quinoa, such as the low cost of its production, its ability to adapt to extreme and varied conditions, and mainly its nutritional value. Quinoa can offer a rich and nutritional diet that is low cost, especially in areas where growing other nutritious crops might not be very feasible. This creates a conundrum between balancing the safety (regarding nutrition, economic, and environmental sustainability) of indigenous regions, and the improvement of food security across the world.

This review article reports the outcomes of current research regarding the nutritional and biological prosperities of quinoa, which has been produced in different parts of the world. Moreover, considering the importance of post-harvest processing in preservation of the nutritional attributes of quinoa, extension of quinoa seed shelf life and its products, and the development of various quinoa food products, the following section of this review focuses on the traditional and new technological processes which have been used for post-harvest processing of quinoa as well as side stream processing of by-products of quinoa production and processing. These methods come with consequences, and as such the last part of this paper reviews the emerging issues around marketization choices, social, and ecological features that can follow. The aim is to provide a complete review of quinoa’s production and consumption ends that will help rationalize the decision-making process around its use.

## 2. Materials and Methods

A literature review was conducted with the aim to map quinoa cultivation and to explore the nutritional analysis in different regions from the last 20 years. The initial screening of articles was performed with the University of Hohenheim search engine (https://rds-hoh.ibs-bw.de/hohsearch/) that gathered different databases and single journals provided by the university. Additionally, external sources were handpicked from the references list of the referred articles. The FAO/INFOODS Food composition for Biodiversity (BioFoodComp v 4.0-2017) database was also used as a source of data for the nutritional composition of quinoa. In total, a number of 34 sources were used to form the collection of presented data.

Moreover, a systemic literature review was undertaken to analyze the post-harvest technology innovations in recent years. Academic databases and search engines (e.g., Scopus and Google Scholar) were utilized to find articles in English focusing on post-harvest technology, biological, and nutritional properties of quinoa, traditional uses of quinoa, product development of quinoa based products. Keywords searched included, but were not limited to quinoa products OR quinoa product development as well as quinoa followed by the keywords for the subtopics. For post-harvest technology: production; post-harvest: processes; evolution; innovation; for biological and nutritional properties: nutritional properties; biological properties; bioactives; superfood; for traditional uses: traditional consumption; traditional products; traditional food products; for product development of quinoa based products: innovation; trends; food trends; future products; new products.

In addition, a comprehensive literature review was conducted in order to better analyze side stream processing methods of quinoa waste and the potential for byproduct utilization in various commercial industries. Databases and search engines (e.g., Scopus and Google Scholar) were utilized in searching for articles in English pertaining to the topic between October and December 2019. Keywords utilized for the search included: biogas AND quinoa waste AND alternative use AND biomass yield AND pellets AND processing AND antibacterial AND husk AND saponin AND topical application AND quinoa extract AND starch granules. As a result of these searches 13 articles were explicitly referenced regarding this area. These articles were relevant to the aim of this paper due to their comprehensive coverage of quinoa usage in feedstock, substitution for conventional fuels, and as a novel molluscicide.

Furthermore, to provide a perspective on the environmental and social impact of the increasing quinoa demand, a systemic literature review was undertaken. Academic databases and search engines (e.g., Scopus and Google Scholar) were utilized in searching for articles in English in two distinct periods, before and after 2014, focusing on the peak of the quinoa boom and its aftermath. The search was divided into two main topics: consumption and production; and other four related sub-topics, namely welfare, nutrition, environment, and market. The main keywords included: (consumer OR consumption and producer OR production) AND (quinoa) following the keywords for the subtopics. For welfare; (income OR household OR welfare OR prices OR impacts); for nutrition (diet OR nutrition OR food) AND (accessibility OR security OR impacts]; for environment: (impact OR sustainability) AND (biodiversity OR environment OR diversity OR conservation); and for market (market OR economy).

Last but not the least, a critical analysis of the quinoa market was conducted to develop a qualitative outlook and to provide projections regarding this market. The database selected comprised of Scopus, Google Scholar, and Science Direct, where recent articles published in English were perused to understand recent market trends on quinoa. This was further merged with a keyword search that included (Quinoa market OR Hybrid Economy OR producer and consumer welfare OR Outlook, Neo-liberalism OR Sustainability OR Functional food product development). Guidelines were followed to exclude studies out of relevant and intended market research bounds. As a result, in accordance with the keywords, 13 articles were chosen to critically analyze the quinoa market and to produce an outlook on market trends. 

## 3. Nutritional and Biological Properties of Quinoa

The exceptional nutritional value of quinoa relies on its balanced composition of high protein, amino acid profile, minerals, fibers, and minor compounds (such as antioxidants and vitamins) [9]. Moreover, due to the absence of gluten, quinoa is suitable for celiac patients or gluten related disorders. Several factors may affect the nutritional composition of quinoa seeds and the yield of the plant. Genetic and environmental conditions are two factors that may affect the yield and nutritional quality of quinoa. Accordingly, quinoa cultivation altitude can range from sea level to 4000 m high, and cultivation location ranges from Colombia (2° N) to Chile (47° S) in its origins. This variability in cultivation location and altitude, as well as rainfall regimes, has led to a high biodiversity of quinoa species, given that growing conditions are different for each location and thus plant adaptation was required [1]. Moreover, quinoa breeding programs are focused on developing high yielding varieties with desirable nutritional properties which are better environmentally adapted to several agroecological zones. Emphasis is placed on the consumer markets—namely rich westernized countries—as quinoa has gained recent attention as a ‘superfood’ [11].

### 3.1. Proximate Composition

The proximate composition of quinoa seeds, as reported in the literature, is presented in Table 1. Among the macronutrients, carbohydrates can be found mostly on the perisperm of quinoa seeds, while the endosperm and embryo are richer in protein, minerals, and fats [1,11].

Briefly, a thorough assessment of the reported data regarding the nutritional composition of quinoa by Nowak et al. presenting the data from 27 articles (103 data lines) found considerable variation of nutrient values among different varieties from different locations [17]. Values reported in g 100 g^−1^ edible portion—Fresh weight basis ranged as follows: protein (9.1–15.7 g), total fat (4.0–7.6 g), and dietary fiber (8.8–14.1 g) while the moisture content of quinoa is reported to be around 15%. In their report of the data, the majority of entries (68) were from samples from South America—mainly from Peru and Bolívia (the biggest producer of quinoa in the world)—followed by data from Europe (23) and Asia and North America (six each). This reflects the traditional production of quinoa in South America but also the expansion of its production worldwide [17].

#### 3.1.1. Protein and Amino Acid Content

The protein content of quinoa seeds ranges between 11% and 19% (Table 1). Moreover, quinoa seeds contain all nine essential amino acids (EAA) for proper human health as noted in Table 2 [19].

Quinoa has garnered attention as a protein source due to the high quality and balanced composition of amino acids content of its protein—superior to wheat, barley, and soybean. Quinoa essential amino acid scoring patterns (Scoring patterns, as defined by FAO, are based upon on the amino acid requirement values divided by the mean protein requirement [20]) can be seen in Table 3, which shows quinoa exceeds the scoring patterns for 8 essential amino acids [20,21,22]. 

The appreciation of quinoa as a food by Andean populations relies on its high nutritional value, as it is the principal protein source for rural populations, substituting the lack of animal protein [23]. Moreover, due to its high protein content and amino acid profile, quinoa is suggested to be an alternative to dairy products [23,24].

The protein and respective amino acid profile of quinoa can vary significantly from cultivar and location (Table 1 and Table 2). Quinoa can be grown on various types of soils; nevertheless, the plant responds well to nitrogen fertilization, increasing yields, and protein content of seeds. The application of organic matter is important for topping nutrients and promoting water use efficiency in arid regions and sandy soils, thus enhancing the seed yield [25].

#### 3.1.2. Carbohydrates

The carbohydrate content of quinoa seeds ranges between 49% and 68% (dry matter weight) (Table 1). Starch is the main biopolymer constituent of plant organs, and is the most abundant carbohydrate present in the seeds. Native quinoa starch consists of uniform small granules less than 3 µm in diameter [24,26]. Quinoa starch also presents interesting functional applications, due to its low temperature of gelatinization (range of 54–71 °C) and enthalpy (11 J g^−1^ starch) [12]. Compared to the starch of wheat and barley, quinoa presents a higher maximum viscosity, water absorption capacity, and greater swelling power [26]. Its excellent freeze-thaw stability makes it an ideal thickener for food products where resistance to retro degradation is desired [12,26]. Additionally, due to the small-sized granules and high viscosity, quinoa starch has the potential to be used in specialized industrial applications, such as dusting starches in cosmetics and rubber type mold release agents [24]. 

Another carbohydrate group present in quinoa seeds is dietary fiber. The total dietary fibers content of quinoa seeds is close to what is found in other cereals ranging from 7.0% to 9.7 % (DM) [26]. Pulvento et al. reported an average of 17.2% of dietary fiber in quinoa harvested in the south of Italy. Although representing a high content, dietary fiber can decrease significantly after post-harvest processes to eliminate anti-nutritional micro components present in seed coats [13]. Table 1 notes the fiber content of quinoa found in the literature.

#### 3.1.3. Fat

The fat content of quinoa seeds varies between 2 and 9.5%, which is higher than maize and other cereals but less than soybean (Table 1). Quinoa oil is rich in essential fatty acids such as oleic [C18:1] (19.7%–29.5%), linoleic [C18:2] (49.0%–56.4%), and linolenic [C18:3] (8.7%–11.7%). The portion of (poly-) unsaturated fatty acid accounts to 87%–88% of total fatty acids of the seed [12,26]. These compounds have gained importance since they promote health benefits such as positive effects on the immune system, cardiovascular diseases, cell membrane function, and increased insulin sensitivity [18,26]. Table 4 shows the reported results of determining the fatty acid content and profile of quinoa seeds of different varieties cultivated in different locations. Quinoa may also be considered an alternative oilseed. The oil contains a high concentration of antioxidants such as α- and γ-tocopherol, which ensures quinoa oil a long shelf life due to its natural antioxidant potential at the level of cell membrane, protecting fatty acids against damage by free radicals [26].

### 3.2. Micro Components

Distributed across the macro components of quinoa seeds are micro constituents such as minerals and bioactive compounds are present in minor scales. Such micro constituents contribute to not only the nutritional composition of quinoa but also may be used due to their functionality. Moreover, the exceptional nutrient profile from quinoa can provide valuable therapeutic properties such as enhancing immune function, assisting in cell repair, calcium absorption and transport, participation in the metabolism of fatty acids for human health, and even preventing cancer metastasis [19,26].

#### 3.2.1. Minerals

As it can be seen in Table 1, the ash content of quinoa seeds ranges from 2.4% to 4.8%. The ash contains a diversified profile of minerals including a high content of calcium, magnesium, iron, copper and zinc. The mineral content of quinoa seeds is found to be at concentrations greater than most grain crops [25]. Table 5 shows the mineral content of quinoa and its comparison with other grains [21,22].

Vega-Gálvez et al. reported that mineral concentrations seem to change drastically when quinoa is cultivated in different soil types—thus with particular mineral compositions—and fertilizer application [25]. Table 6 summarizes the reported values for mineral content of quinoa seeds reported in the literature.

#### 3.2.2. Bioactive Compounds

Quinoa seeds are the main edible part of the quinoa plant, nevertheless quinoa leaves are rich in phenolic compounds that present antioxidant and anticancer properties. Plant polyphenols and phenolic content are beneficial to human health, due to their antioxidative potential. It has been suggested that such compounds can aid the risk reduction of cardiovascular diseases, neurodegenerative disorders, and diabetes [29,30].

Considerable amounts of ferulic, sinapinic, and gallic acids, kaempferol, isorhamnetin, and rutin were obtained in quinoa extracts. These named compounds were linked to an inhibitory effect on prostate cancer cell proliferation and motility [30].

#### 3.2.3. Saponins

Saponins are grouped among the minor components, secondary metabolites, broadly studied due to their biological properties. They are considered to be the most anti-nutritional factor in quinoa seeds, acting as a natural protection against pathogens and herbivorous. Over 30 types of saponins can be found distributed in quinoa plant parts [31]. The quantification of saponin content is important in order to differentiate between ‘sweet’ (having saponin content of 20–40 mg g^−1^ dry weight) and ‘bitter’ genotypes (>470 mg^−1^ dry weight) [13]. The saponin content found in quinoa seeds reported in the literature is presented in Table 7. Saponins confer the bitter taste and are mostly found in the outer seed coat. The compound is removed by post-harvest processing techniques like cold water washing, abrasion, and dehulling [19]. In addition, saponins extracted from quinoa seeds can be used in other industries such as cosmetics and pharmaceuticals.

#### 3.2.4. Vitamins

Quinoa is also a source of vitamins, namely riboflavin and folic acid, offering similar values of thiamine, but is a lesser source of niacin. It has been noted that the removal of the saponins (to reduce the bitter taste) does not seem to affect the vitamin content [21,22]. Vitamin content of quinoa and compared to the other grains can be seen in Table 8.

## 4. Post-Harvest Processing of Quinoa

Immediate post-harvest processing of quinoa includes drying or stacking, threshing, venting, and storage. Drying or stacking involves arranging the plants in stacks immediately after cutting and can be performed in different methods, including Arcos (stacking in crosses), Taucas (panicles ordered towards the same side), or Chucus (cone shaped mounds) [6,32]. Threshing consists of the separation of grains from the panicle. It can be done manually, semi-mechanically (tractors or trucks are used to pass on top of the panicles, a yield of 1000 kg/h is obtained), or mechanically (similar yields as semi-mechanical, around 1000 kg/h but easier to process in the following steps) [6,32]. Winnowing consists of the separation of ‘Jipi’ or perigonium and biomass from the commercial grain. In this step, three main methods are used: (1) a traditional method that is dependent on the presence of moderate winds with low yield of about 400 kg/day, (2) an improved manual method which uses a prototype machine that regulates air flow with yield of 600 kg/h, and (3) a mechanical method which uses a designed machine for the winnowing that has a yield of 500 to 800 kg/h that is higher than the yield of the two mentioned methods [6,22]. Finally, the last step is storage of grains before further processing them into other food products. Grains should be stored in woven llama wool, or in polyethylene bags [6,32].

Further processing of quinoa seeds is needed to obtain grains that meet quality standards in terms of size, impurities, or extraneous material. The grains undergo a series of processes including: preliminary sorting and removal of impurities; saponin removal; drying; sorting by size; separation of grains based on their color; and removal of residual impurities [6,32].

Moreover, quinoa production has been steadily increasing over the past few years, and is projected to continue to increase over the next few years [8]. As a consequence, various industrial-scale innovations have been introduced for the harvesting and postharvest steps to replace the traditional practices that were initially conceived for small-scale production [32]. 

As exemplified by the Bolivian example the quinoa production chain has three main sections: the agricultural production of the grain, its processing, and the production of value-added products. Furthermore, saponins, a hindrance to the consumption of quinoa but an important added value resource, were not being recovered from the processing of the grain. To combat this problem, a program was created, named the ‘Quinoa Alliance’ [33]. It focused on developing the second chain in the production of quinoa, the processing of the grain.

Quinoa production is still small, which explains why specific machinery was not being developed to help resolve the technological limitations that hinder the grain processing and why the market for such technologies was limited. Companies resorted to the use of adapted technologies, which in turn resulted in efficiency problems along the processing chain. 

An example is quinoa scarification, which was being conducted in a peeler that was originally designed for rice scarification. In brief, the peeler uses friction and rubbing mechanisms while the quinoa grains are raining against a metallic net to ensure saponin removal. Adjustments were made to speed up the process, through friction between grains [33]. After finding this problematic example, a deeper study was conducted on all of the post-harvest processing steps. As a consequence, a few inefficiencies were found: there were significant losses of raw material, diminished quality of the grain, increments in production cost due to high specific consumptions of water, electrical energy and gas, high operational costs due to the use of labor force, and production of wastewater residue with high contents of saponins. It was determined later that these inefficiencies led to the pollution of bodies of water as well as made the recovery of pure saponin infeasible, with the consequent loss of its commercial value. Table 9 shows a summary of the cause of inefficiencies in quinoa processing and the solutions that have been adopted to make postharvest processing of quinoa seeds more competent according to the reported results of the ‘’quinoa alliance‘’ program [33].

As stated in Table 10, with the development and implementation of such new technologies, processing capacity was drastically increased [33]. 

## 5. Food Products and Beverages Originated from Quinoa

### 5.1. Traditional Food Products and Beverages Originated from Quinoa

Table 11 shows a range of traditional food products prepared with quinoa and their brief description.

As shown in Table 11, typical traditional uses of quinoa relied essentially on its use either as a grain or as a flour, which was then used to make different breads, soups, and fried or cooked products, with few products using quinoa leaves as an ingredient as well. Respectively, it can be stated that among the earliest uses of quinoa as food product is its consumption as a simple seed, which can be considered as the staple traditional quinoa product., Traditionally speaking quinoa flour was obtained through grinding (‘aku jupa’). In order to obtain suitable organoleptic characteristics, a mixture of different flours might be needed (e.g., a mixture with rice flour) [1,6,32].

Expanded/puffed quinoa is another quinoa product that has been consumed for several millennia. The pearled grain is subject to high temperatures and pressure, and then forcibly expelled, thus causing a sudden pressure drop that makes quinoa pop, releasing its internal moisture in the form of vapor, and therefore expanding into a light product. Puffed quinoa can then be consumed directly, or grinded into an instant product, as an instant cereal, or as an ingredient of energy bars. The consumption of puffed quinoa however has some drawbacks. Puffing process may contribute to the loss of protein, oleic, and linoleic acids. The adverse effect of the puffing process on the nutritional quality of quinoa may be due to exposure to high temperature and long processing times [1,6,32]. 

### 5.2. Novel Industrialized Food Products Developed from Quinoa

The rise in the production and consumption of quinoa led to the development of new industrialized quinoa products. As mentioned previously, quinoa flour is among the products that are developed from quinoa seeds. Showing its versatility, quinoa flour can be used in almost all products manufactured by the flour industry (such as bread, pasta, sponge cakes or biscuits). In particular, quinoa flour can be used to produce quinoa noodles. Using quinoa flour for noodle production offers an alternative for those who suffer from celiac disease or sensitivity to gluten, as well as creating a product with different organoleptic characteristics than normal noodles. At this point, there is not yet a defined recommended quinoa cultivar that is more appropriate for use in the noodles and pasta industry [1,32,34,35].

Furthermore, quinoa flakes can be considered as one of the new quinoa products that are produced by the combination of drying (to 15% moisture) and pressing process between two converging rollers. Studies have shown that the final characteristics of the flakes will depend on the variety of quinoa, as the plasticity characteristics of quinoa will vary with different quinoa [32]. After obtaining quinoa flakes, these can be used in a wide range of applications in other food products (e.g., juices, soups, pies, or cakes), as they require less cooking time than other grains [32]. Another option is to create expanded quinoa products (or *pisankalla*), made from the pearled grain [32], as mentioned previously.

In addition, quinoa based extruded products developed during the past years can be noted as new quinoa food products. Quinoa extrusion processes involve high pressure and temperatures, as well as shearing over short periods of time. This process restructures the starch and protein content of quinoa, thus producing different textures. The physicochemical reactions during extrusion processing of quinoa involves the following: a) starch gelatinization and dextrinization, protein texturing, and partial denaturation of the vitamins present; b) melting and plasticizing of the food; and c) expansion by flash evaporation of moisture. Pearled quinoa is fed into an extruder, undergoing a machine and thermal transition to form semi-solid dough. The dough is then extruded through the machine and sheared at the outlet with a rotary cutter to obtain the final product [32]. In contrast to the puffed quinoa, there is no apparent nutritional degradation of quinoa in this process, probably due to shorter processing times [32,34].

### 5.3. Other Products Derived from Quinoa

In addition to the above mentioned products, there are some other potential products derived from quinoa. Such products are obtained through the extraction of compounds from quinoa and for creation of added value products. Examples of these products include oil extraction, protein concentrates and isolates, starches, bioactive compounds, among others.

Quinoa contains a high protein content (see Table 1) and can be used to produce protein concentrate. To obtain a quinoa protein concentrate, a more complex process is used. Firstly, the fat-free germ or embryo has to be isolated. Then, it goes through a high temperature alkaline extraction (pH 11.5 at 50 °C), followed by centrifugation, washing, and centrifugation again. The solid residue resulting from this process is then precipitated at pH 4.8 (isoelectric precipitation), and centrifuged once more. The solid matter is again washed and centrifuged and vacuum dried (30 °C), to finally obtain a protein concentrate with the desired functional properties and characteristics [1,32,34,35].

Quinoa’s carbohydrate content is consisting 54% of small granular starch (0.6 to 2 µm). Although processing does not significantly improve the digestibility of starch, it does affect binding and organoleptic characteristics of the final product. In this respect, compared to starch content of wheat and barley, quinoa starch shows higher viscosity, higher water retention, and expansion capabilities as well as higher gelatinization temperatures, resulting in better performance as a thickening agent. On account of such properties, quinoa starch is extremely appropriate for the production of prepared frozen baby food as it shows good freeze-thawing stability. However, its properties are not as competent as those of wheat and barley for preparing quinoa-based breads and cakes, mainly due to the lack of gluten [1,32,34,35]. Lastly, based on its protein and starch content, quinoa may be used for production of edible films as well as an emulsion stabilizer agent, namely for Pickering emulsions [34]. Quinoa incorporation in food products has been proven to help extend shelf life and to reduce microbial spoilage of food products. An increase in shelf life of quinoa food products specifically at the structural and organoleptic level could be due to a lower degradation rate of starch molecules. When taking into consideration the presence of bioactive compounds such as polyphenols, quinoa can assist the microbial preservation of food products, such as the inhibition of mold growth [1].

## 6. Side Stream Processing of Waste of Quinoa Production and Processing: Potential of Quinoa Applications

### 6.1. Side Stream Processing and Utilization of the Quinoa Husk and Saponins

It can be stated that the most significant innovations in quinoa processing are related to the saponin removal unit operation. As mentioned before, saponins must be extracted from quinoa due to their bitter taste, in order to obtain a quinoa product with desirable organoleptic properties. However, through the recovery of saponins from quinoa, it is possible to create a viable by-product that can be used in other industries. Other polysaccharides that can be extracted from quinoa have also been reported to show biological activities, thus presenting other possible revenue streams [1,36,37]. 

Due to the physicochemical and biological properties of saponins, these can be used in several commercial applications in the agricultural (e.g., as a bio-insecticide), food, cosmetic, and pharmaceutical sectors. For example, based on its foaming ability at low concentrations, it can be used mainly in the food and cosmetic products. Despite the fact that saponins are being considered as an antinutritional substance, there are also studies of their positive health effects due to their anticarcinogenic, anti-fungal, and cholesterol lowering effects, which are of great interest for the pharmaceutical industry [6,30,32,35,38,39,40,41]. In this respect, various studies have shown that alkali transformation can be useful in transforming certain properties of phytochemical such as quinoa saponins [42]. Sun et al. investigated the effect of saponins extracted from quinoa husk on oral bacteria. They investigated the antibacterial effects of the quinoa saponins that have been transformed by an alkali treatment on three types of halitosis-related bacteria: *P. gingivalis*, *C. perfringens*, and *F. nucleatum* [43]. The outcome showed that the different concentrations of alkali- transformed saponins showed significant inhibitory activity against the bacteria, when compared to the primitive quinoa saponins. The researchers conclude that the less polar saponins have the ability to easily interact with, and damage, the cell membranes of the bacteria. Moreover, among the advances in developing novel molluscicides against the Golden Apple Snail (GAS) is using the quinoa husk [43]. The GAS is considered to be one of the most invasive species in the world and has threatened rice agriculture in many countries. The benefits of using the quinoa husk instead of a synthetic molluscicide are that it does not show any toxicity to fish that are included in rice production at the highest concentration tested [43]. It was noted that the quinoa-based molluscicide killed 100% of the Golden Apple Snails in a laboratory setting, showing that it is effective at high doses without harming fish. In this regard, Joshi et al. followed up on these results by investigating the extent to which the quinoa saponin product protects new rice seeds from the Golden Apple Snail (GAS) [44]. The outcome of their investigation showed that the GAS mortality of 94% can be achieved by the application of 11 ppm quinoa saponin in 48 h, while it has not predominantly affected the growth of rice shoots [43]. Therefore, quinoa saponin may be used as an environmentally friendly alternative for protection of rice cultures against GAS.

### 6.2. Side Stream Processing and Utilization of the Quinoa Biomass

Quinoa also has the potential to be an alternative source of energy when utilized as a substitute for conventional fuels in rural areas in the form of pellets. For production of pellets, the biomass of crop is burned to produce ash, which is then combined with starch. In certain rural regions of Peru, the access to modern energy sources is still limited and farmers utilize agricultural waste as an energy source to cook their food [45].

The usage of quinoa as feed for animals is another expanding area that is highly dependent on the amount of residue. Quinoa biomass can be utilized as fodder in areas where other crops cannot grow at elevated altitudes. Quinoa husk and seed bran are also potential sources of feed. It is specifically the dry matter yields that show the most potential given the protein amount and digestibility possibility [4].

Quinoa grain bran consists of the hull of the grain and the pericarp [4]. According to Paredes et al. farmers utilize the bran for feed or composting purposes and investigated the addition of 30%–60% bran to feed guinea pigs [46]. The obtained results concluded that 30% quinoa bran has the potential to be substituted for normal feed consumption with a beneficial effect regarding anti-parasitic effects from the quinoa by-products. Moreover, when quinoa by-products are utilized for feed of non-ruminating livestock, studies have concluded that the proportion of quinoa must not be greater than 30% of the diet [4]. For ruminating livestock, the leaves and stalks from the harvest can be used without having concern over saponin presence. The usage of quinoa by-product for feedstock has potential, but more research needs to be conducted in this area, taking into account the costs and the competitions that may arise in acquiring by-products that contain saponin based on its application in multiple industries (cosmetics, pharmaceutical, etc.).

## 7. Production, Marketization and Consequences

In this section we review how quinoa’s production and marketization is organized so far in the light of global trends that will affect the market heavily in the next decade. In order to do so, we have to contextualize quinoa’s production in the current global environmental, social and market situation. Climate change has created disparity amongst various dependent entities such as wildlife, forestry, marine, and humanity itself. Consequently, a change in diet has also been emphasized by the UN and the Intergovernmental Panel on Climate Change (IPCC) as a section of their special report in 2019, as a way to reduce the land clearing and the consequences of animal agriculture [47]. The emphasis has been laid on consuming a plant-based diet and to lessen meat consumption. It should be noted that when disposable income rises up, individuals consume more meat as a result of cultural norms and marketing for profiteering [48,49]; therefore, a good alternative to meat and its environmental impact has to be provided if we want to meet the environmental and health-related needs.

Secondly and consequently, the market has seen an increase in consumer awareness [50,51]. The demand for healthy, environmentally friendly, and safe products has increased and there is agreement that in the next decades these influences will continue to rise. It should be noted that alongside this, food insecurity is still present and often plagues areas long exposed to malnutrition, as well as locations where economic activities still strongly revolve around agriculture.

Lastly, the Euromonitor [50] has highlighted five top trends in regard to changing food markets: (1) ‘free from’ foods such as dairy-and gluten-free foods being considered as the most dynamic in health and wellness [50,51,52,53]. ‘Free from’ foods alongside plant-based diets are crucial factors in reducing meat consumption; (2) the rising demand for natural foods such as organic, although the definition of what organic stands for varies across borders and causes debates [53]; (3) functional foods, which are value-added products that provide additional benefits to one’s health in comparison to normal nutritional attributes of a food product [54], which are on the rise in emerging markets such as in the Asia-Pacific, targeting nutritional gaps, but decreasing in Europe as consumers perceive them as artificial and processed [50]; (4) the energy-boosting foods, included in a holistic diet that enables physically fit consumers to maintain longer durations of energy (for instance paleo and keto diets) [50]; (5) paradigm shifts in packaged foods shopping habits are leading to subscription meal kits that focus on ‘Health and Wellness’ (HW), a niche whose growth factor is faster in comparison to conventional packaged food. For instance, in the US the percentage of adults looking for added minerals and vitamins in their meals has reached 65% [51].

Where does quinoa stand in this? It seems to be perfectly positioned for meeting contemporary expectations and needs as was just highlighted above, due to its agronomic characteristics, nutritional value, health benefits and by-product use that we exposed in the first part of this article. It is easy to see, then, that for its characteristics and in response to the global trends above mentioned, quinoa, considered a functional food, has consequently seen a peak in demand. Despite the seemingly positives coming from an increasing market demand, some issues have risen because of the nature of the product and the consequences that quinoa’s production and marketization have.

According to the thorough research conducted co-jointly by FAO and CIRAD [54], the largest producers of this crop are Bolivia and Peru (80% market share) and the production has been boosted up by 300% between 1980 and 2011, followed by other countries where this product is not indigenous [54]. In these two aforementioned countries, this crop has been known for centuries for its resistance, and the conditions of production are similar, since the environment in the southern Altiplano is characterized by “extreme conditions—rocky or sandy soil almost permanently exposed to drought, frost, El Niño events, violent winds and intense solar radiation due to the high altitude” [55] (p. 364). In addition, in these original places agriculture is a complex family or community activity, which also helps explain the commercialization decisions of the peasants. These decisions include never abandoning other crops despite the big success of quinoa internationally, and intermixing husbandry (llamas above others) as this seems to have a role in how this crop is produced. Quinoa is (was) their staple food, and it is strictly linked to identity and stability [55]. However, this has not prevented quinoa from becoming decontextualized, as we will see in the next paragraphs. 

Regarding foreign production, the FAO and CIRAD report [54] has provided a comprehensive overview for European countries. In France, it was decided to pursue locally produced “Quinoa d’Anjou”, in the Loire area, during the period 2009–2012 [54]. After only a period of three years, the sector organized itself with conventional producers; and still, commercialization and slow market entry was also observed by the agri-food industry. In Italy, tests have shown that quinoa can be cultivated in the southern regions and would flourish under adverse conditions from nature as well. Italy is also predicted to have a shortage of water in the near future (alongside a quality deterioration of water and other forms of abiotic stresses, as a result of climate change). Turkey is also considered to have potential regions capable of producing quinoa [54]. With arid and semi-arid regions, the country also has a large population to feed and has to deal with abiotic stresses, due to climate change which may lead to problems such as lower crop yields. It is not a customary practice to cultivate quinoa with full irrigation. Drought is one of the deadliest environmental issues facing the country alongside social and economic problems where farmers have higher input costs and suffer low crop prices. In fact, in the case of Turkey, the results are different and show a positive response with full irrigation both under freshwater as well as saline water [54]. Other areas such as Morocco, Greece, and the Indian subcontinent (India and Pakistan) have also conducted positive tests, and India particularly has shown interests in developing their own markets for quinoa. A common problem these countries face is a system that has a weak market or lack thereof, risk-averse behavior shown by farmers, and a major lack of knowledge and technical diffusion. In conclusion, the tendency to test this crop according to the local production’s conditions to expand national markets is on the rise. Consequently, along with a rise in production, we have seen significant increases in prices, which have tripled from 2006 to 2013. Since quinoa production became competitive, low tariffs were introduced in EU and US with intense marketing and eco-labeling, with a direct consequence of a price increase [54]. Since demand and prices are annually spiking with instability, new producers have emerged in the open market economy in the Andes with conventional methods of production. These methods are not deemed sustainable for the environment [56]. Such high demand has also led to international conflicts such as the smuggling of quinoa across borders. This was documented when the army of Bolivia incinerated quinoa produce worth 23 tons that had been smuggled from Peru into Bolivia; and was meant to be sold as organic products [48].

### 7.1. Socio-Economic and Environmental Issues Related to Quinoa Cultivation and Marketization

Currently, quinoa as an export product has mainly reached the educated, medium-upper class consumers, who are open to trying new food products and fitting the food trends mentioned above. However, the original consumers are the peasants for which quinoa has been a staple food for ages, and is embedded within their traditions and customs [50,51,52,53]. Global North consumers are the target for this product as the profit margins are very good. Competitive marketing using social media networks and technological advances came as a response to healthy food trends. Demand also increased for culturally rich and ethical food that comes from ancestral cultures and natives, mainly ones who produce organically in most cases and, unsurprisingly, also experience poverty. In fact, during the peak of the quinoa boom in 2013, media coverage shed light on the negative effects of the increased demand of quinoa on the welfare of its producers in the Andean region [8,57,58]. These articles alarmed consumers about the grave consequences of rising quinoa demand for the producing Andean countries such as: loss of biodiversity, conflicts over land, and dietary changes (including westernization of diets and decreasing consumption of quinoa). Aside from quinoa, Blythman (2013) turns to other examples from the Global South, such as the case of Peruvian asparagus and the impacts of soybean production in South America [57]. Further parallels with quinoa will be drawn in the section below, pointing out other crops in the Global South-North exchange of food commodities in parallel to quinoa. Along with the public discourse, academia has been raising a range of issues related to the quinoa boom’s environmental and social impact. While studies presenting empirical evidence have been published on diverse topics, critical aspects have been raised with respect to the sustainability of quinoa production. Thus, there is still a big ambiguity present in the discourse. The increasing demand for quinoa coming from the Global North and its future prospects concerning the posed environmental impacts, raises a series of challenges for the cultivating countries in the Global South, especially for the three major producers, Peru, Bolivia, and Ecuador. The calls to action regarding the deteriorating biodiversity mostly touch upon two main aspects, namely: the utilization of the available genetic diversity of the crop and its relation to market pressure and the effects of changing land use on biodiversity. The genetic diversity of quinoa is connected to centuries of work of traditional cultivars, passing on the knowledge from generation to generation. The crop’s wide genetic diversity and adaptability are key characteristics of quinoa’s growing importance in the light of today’s global challenges (e.g., climate change) [8]. However, the loss of genetic variety does not only have an impact on biodiversity and producers, but it includes nutritional aspects as well [59] (p. 115). Studies have identified and analyzed the reasons that producers opt for certain quinoa varieties. In the context of Peru and Bolivia, Carimentrand et al. emphasize the different tendencies of selling mixed quinoa seeds on local markets, while simultaneously responding to the international and urban demand for standardized products as “agro-industrial companies and exporters seeking to meet the market demand for uniform and large grains, encourage producers to sow improved quinoa varieties” [60] (p. 331).

Astudillo and Aroni in 2012 gathered quantitative and qualitative data in southern Bolivia (namely, Salinas and Colcha K municipalities) regarding the use of quinoa varieties. It was observed that “Of the quinoa produced in the study communities, 90% came from only five of the more commercial types or cultivars (out of the 40 types found in the area)” [59] (p. 106). Moreover, the percentage of people who cultivated only one type of quinoa significantly grew and the research also highlighted socio-economic determinants, which were influencing factors in choosing the use of a certain variety. While this regression analysis highlighted influencing determinants as “the number of varieties a farmer grew in the past, membership in a producer association, the area planted of quinoa and father’s education” [59] (p. 111), focus groups shed light on more aspects such as marketability, cost of production, land size, limited access to seeds, preference for sow seed mixtures, and considering certain types as “lucky” and “unlucky” based on yields [54] (pp. 114–115). In Chile’s main quinoa producing areas, a wide genetic variety had been identified with microsatellite analysis, and a field survey was conducted to analyze the management practices of local varieties [61]. Fuentes et. al. pointed at the need to prevent the loss of biodiversity due to increasing market demand and pressure for homogenized seeds [61]. On the other hand, Winkel calls out the alarming voices raised about endangered biodiversity and highlights studies which “suggest that the booming commercial production in the southern highlands of Bolivia has not altered its biodiversity” [62] (pp. 96–97). In the *State of the Art Report of Quinoa in the World in 2013* the authors explain the boom’s lack of “appreciable” impact on the genetic diversity as: ‘’(i) different kinds of quinoa have continued to be used locally for a wide range of food preparations (Table 11), as well as for medicinal and ritual uses; (ii) the commercial product, ‘Quinoa Real’, is identified with a set of diverse varieties which were traditionally cultivated and which have now found a market: white grain quinoa, dark grain quinoa, and quinoa for puffed grains’’ [63] (p. 365). 

A further key aspect related to the debated environmental impacts, which incorporates the challenges to biodiversity as well, is the change in land use. This includes among others, the increasing area under cultivation, the phenomenon of farmers opting for mono-cultural agriculture and the replacement of traditional agricultural methods. In detail, regarding land use change, Chelleri et al. notes a “sharp growth in the percentage of plots dedicated to quinoa mono-cropping in 2012 compared with 2010 and 2008” in the communities studied in Tomave municipality, Bolivia [64] (p. 2233). In Peru changes in the land use in relation to the international quinoa market had been analyzed by Bedoya-Perales et al. in the framework of three phenomena, namely displacement, the rebound effect and the cascade effect [65]. The study notes the expansion of land acreage both in traditional and new producing regions. Thus, as yields increase, it can lead to changing traditional agricultural methods, expansion of land and new technologies. Bedoya-Perales et al. note the environmental impacts (e.g., loss of genetic biodiversity and “the emergence of difficult-to-control pests”) of the quinoa boom related to the cascade effect in traditional producing areas, such as in the Puno region [65] (p. 9). Latorre Farfán in 2017 analyzed in the framework of sustainable production in Peru’s two main producing regions: in the area of Majes, Arequipa region, and its conventional intensive production of quinoa, and in Camacani in Puno region, where traditional methods are in use. Putting focus on Majes, the study highlights the need for utilizing the resources more efficiently as one of the pillars to develop sustainable farming systems [66] (p. 47). Changes in land use and its cause and effect relations are a deep-rooted and complex challenge present in the sustainability discourse and it is no different in the case of quinoa. As the current academic discourse illustrates, understanding present land use change on the local level is necessary in order to improve future forecasts and to achieve a more sustainable agricultural production. At the same time, from local peculiarities in climate conditions and production methods, challenges can arise for pursuing a meta-analysis and setting the research and policy agenda accordingly. Moreover, local-global linkages have to be taken into consideration, too, such as the relationship between land use change-related negative externalities and international market prices and the integration of traditional knowledge with the state-of-the-art research on sustainable agricultural production. Mechanization of agricultural practices are an additional characteristic connected to changing land use. Alongside positive effects (i.e., increased productivity), negative consequences can arise from mechanization, too. For example, in the framework of land management, Carimentrand and Ballet underline the connection between the arrival of tractors and land clearing as form of land appropriation, having a considerable impact on land ownership patterns in the Southern Altiplano of Bolivia [67]. Connected to the appropriation of communal lands, Winkel and colleagues highlight the dynamics between the quantitative patrimonialization of lands, reaching its limits in space, and the qualitative patrimonialization, which is needed in the future in order to “improve cropping practices, and negotiate comprehensive agreements for using common land resources” [68] (p. 201). For this reason, addressing extensive agricultural practices raises the need to tackle the topic of governance of local resources as well [68,69,70]. In addition, soil erosion [71,72] or poor seed placement [72] can be risks of mechanization, too. Walsh-Dilley notes in her study of San Juan de Rosario community in Bolivia, that since the efficiency of using tractors is perceived to be decreasing (e.g., due to quality of new quinoa lands, land degradation, and climate change), producers are opting for returning to hand labor to minimize the risks occurring [72]. 

The challenges to biodiversity and changing land use practices include also the question of livestock, especially llamas, whose manure is used for fertilization. The llama population and its role in sustainable quinoa production can be assessed two-fold. Gandarillas et al. in 2015 notes in Bolivia the shrinking gazing lands and the decreasing number of llama population due to farmers’ switching to quinoa production [73]. Chelleri et al. identified in the study nine communities in the southern Bolivian Altiplano a growing llama population together with an invasion of these animals to the quinoa fields due to the decreased land and other lands of great ecological value [64] (p. 2234). This two-fold aspect sheds a light on the tensions between intensive agricultural practices and sustaining traditional cultivating methods. While on one hand, an increase in production can lead to the decrease of grazing lands for the llama population, thus causing damage for the local ecosystem or quinoa, on the other hand increasing production can also lead to farmers abandoning raising llamas for living resulting a decrease in the llama population. Chelleri et al. also note the decreasing productivity in yields presenting observations on farmers’ perception and from productivity data [64]. This observation was noted by Gandarillas et al. in Bolivia as well, not only in terms of increase of cultivated areas but also in the decrease in yield performance [73]. Bedoya-Perales also observed trends related to changes in land use with data of areas under cultivation in Peru [65]. Possible solutions for protecting biodiversity in these times of increased demand need to be proposed. To answer the challenges posed to the environment, studies have been dealing with evaluating possible solutions for safeguarding biodiversity.

An example as such is Bioversity International’s pilot project of Payment for Agrobiodiversity Conservation Services (PACS) in Bolivia and Peru, which concluded with useful insights regarding the future application of PES in relation to agrobiodiversity [74]. However, further analysis is needed on the implementation of these mechanisms. As Drucker et al. notes “PACS instruments can be designed in such a way as to create incentives to act collectively in order to contribute to the conservation goal and receive rewards. By contrast, it is also possible that PACS schemes, if not appropriately designed, could undermine existing institutions of collective action in poor farming communities.” [75] (p. 108). Martinez et al. analyzed in Chile the role of tourism in rethinking a sustainable alternative to traditional agriculture in line with preserving the quinoa heritage of the territories studied [76]. Winkel et al. analyzed the valorization of biocultural heritage for the inclusion of peasant farming through the case of Chile and Bolivia [77]. Community gene banks have been analyzed as part of efforts reversing the genetic erosion of the crop in Bolivia [78]. While different methods exist on policy and market levels, involving the community itself can be a possible strategic path as well in the future of biodiversity conservation and management, in particular with putting emphasis on involving local stakeholders and encouraging participation. 

In addition, the socioeconomic impacts of the booming quinoa production have been part of the dialogue about sustainability. While in the Global North consumers became increasingly sensitive to the possible impacts of their consumption of quinoa on the producing households in the Global South, studies have been aiming to analyze these assumptions too. The study of Bellemare et al. proved to discredit these worries of ethical consumption with analyzing Peruvian households and the relationship between their welfare and rising prices, both in terms of production and consumption in the period between 2004 and 2013 [79]. Similar results were reported and complemented from an own price elasticity of consumption perspective [80]. Furthermore, impacts on welfare and quinoa as a way out of poverty have also been highlighted in the case of Bolivia by De Arco [71]. 

Regarding welfare, another particularly debated aspect has been the decreasing quinoa consumption in the cultivating regions as a result of increasing prices in the global market. In Peru quinoa is a core part in the populations’ diet and Macedo illustrates within a study conducted in Lima how the usage of quinoa is more prominent in households of lower socioeconomic classification [81] (p. 222). McDonell analyses consumption in Peru before the boom and highlights the fact that it resulted in increased purchasing power, leading farmers to be able to buy new type of food products [82]. Furthermore, the opposite trend taking place in urban settings is also underlined, where the increasing price made it difficult for people with low-incomes to continue purchasing quinoa [82]. Stevens provides an in-depth analysis of household nutrition and rising prices in Peru: these prices are just among the many factors, which can influence consumption trends [83]. Migration to urban areas or the previously mentioned socioeconomic determinants of dietary habits, can be also determining factors in consuming quinoa [81]. Winkel et al. reflects on Jacobsen’s findings about quinoa home consumption with highlighting misleading assumptions, such as the weight of consumed quinoa regarding comparisons with other staple cereals in local diets, consumption trends replacing quinoa with rice and pasta but occurring long before the quinoa boom and underlines national programs encouraging domestic quinoa consumption [84]. 

Community resilience in the face of environmental and market challenges also has to be taken into consideration when analyzing social impacts of quinoa production. The concerns (i.e., extensive agriculture, larger competitors, etc.) faced by cultivating communities provoked diverse responses. Thus, besides the economic gains, it is important to address how the quinoa boom resulted in the cultivating communities developing different strategies in terms of resilience, collective action and empowerment. For example, the community of San Juan in Bolivia is answering the challenges posed by the imperfections arising from the global market by creating cooperative and reciprocal activities in the framework of a hybrid economy [72]. In the times of uncertainty (i.e., in connection to land use) cooperatives can lead a way in the governance of natural resources [69]. As revealed by the Rural Advancement Foundation International (RAFI), “Andean farmers have forced Colorado State University (CSU) to surrender U.S. patent #5,304,718 on ‘Apelawa’ quinoa. The anti-patent campaign that began 14 months ago ended on May 1st when one of the quinoa “inventors” admitted that the patent had been abandoned” [85].

Quinoa has been cultivated in the South American Andes region approximately for the past 8000 years. Many observations based on archaeological evidences supports this thousand-year history and pinpoint several cultures’ role in it, as the Incas or Tiahuanacu [6]. Thus, the acknowledgement of traditional knowledge and ownership, which contributed through centuries to the shaping of the crop’s genetic diversity, calls for the consideration intellectual property rights. Patenting quinoa seeds would put in danger the livelihood of small-scale producers and exporting capacity of the producing countries, such as Bolivia and Peru. One of the key documents related to this question is the Nagoya Protocol adopted in 2010, which “is an international agreement that aims to share the benefits of using genetic resources in a fair and equitable manner, and to support the conservation of biological diversity and the sustainable use of its components” [8] (p. 51). Policies on national level are also directed towards safeguarding this heritage, such as in Bolivia, the national policies and the constitution are directed towards safeguarding the national resources, such as seeds and their germplasms [86].

Connected to international property rights, the International Year of Quinoa (IYQ) in 2013 previously mentioned, found also heavy criticism [84,87]. The indigenous communities’ connection to quinoa is often depicted with preservation and safeguarding, instead of creation, strategically undermining the claim for ownership over quinoa by people from the Andes, or as McDonell puts it: “If the IYQ had thanked the Andean indigenous peoples for inventing quinoa rather than merely preserving it “in its natural state,” this framing would support the case, being made by Bolivia, that Andean nations should have some sort of ownership of quinoa germplasm” [87] (p. 81). In the aftermath of the International Year of Quinoa, ethical considerations by Winkel et al. have been raised as well, calling for a need for further analysis, namely “commercial interests, seed property rights and unbalanced competition between farmers from southern and northern countries” [62] (p. 98). McDonell raises similar questions regarding unbalanced competition and calls for appropriate institutional mechanisms when it comes to traditional food commercialization [87]. The question of local markets and production for export was analysed by Soper in Ecuador, unravelling the motivation of Ecuadorian peasant farmers to produce for export, rather than for local markets [88]. Winkel et al. mention the importance of recognizing local/global connections and short value chains regarding peasant biocultural heritage [77]. Thus, alongside with analyzing quinoa production in the global food supply chain, empowering connections between producers and consumers and enhancing fair local market conditions is needed. Unequal power relations, funding limitations, technological access, and gaps in the legal framework are making access to fair position in the patenting system very difficult to attain for the farmers of the Global South [89,90]. Recognition of traditional knowledge, ownership, benefit-sharing and inclusive innovation continue to stand at the core of the debate over intellectual property rights and quinoa [8,89]. Thus, questions remain difficult to answer and future research and policy considerations are urgently needed. 

McDonell proposes a “miracle food narrative” framework of analysis regarding the quinoa adaptation [91]. This addressed the phenomenon from a discourse perspective, in which quinoa consumption is embedded namely in the narrative of commodifying an ‘indigenous’ and ‘ancient’ crop as a curative metaphor for solving hunger and malnutrition. In this framework McDonell places the International Year of Quinoa “as a self-conscious project to foster and strengthen the sociopolitical webs necessary to consolidate quinoa’s place as the incumbent miracle food” [91] (p. 79). 

In the light of the growing academic literature on international quinoa trade and its environmental and socioeconomic impacts, the fraction between professionals in quinoa trade, which Small describes as a debate around whether to reduce or increase production (“Some critics argue that the major need is to *limit* production in environmentally fragile areas; some defenders argue that the major need is to *increase* production in a sustainable way”) is becoming increasingly relevant [92] (p. 177). Due to the fragmented linkages between macro and micro-level empirical evidence on the impacts of quinoa production, the current status and the future of sustainable quinoa production is ambiguous. Thus, taking into consideration the increasing demand and its foreseen continuing tendency in the future, the interpretation of the strategy contributing to quinoa production’s sustainability has to be re-approached. To give an idea of the level of contestation and possible issues rising, suffice it to say that civil reactions due to distrust in marketers and agro-tech have led a minimum of 29 countries including citizens and organizations to file more than 1300 lawsuits against corporate business and government actions within their respective countries and more than 75% were notified in the United States [93].

Looking at the dynamics of quinoa boom, similar patterns can be discovered in other crops from the Global South as well, such as soybean, a ’classical example of an unsustainable agricultural model’ [94] (p. 397). The top producers today are United States, Brazil, and Argentina [95]. Looking at the history and the expansion of the crop in South America, we can understand how it has become a commodity characterized by Global South-North dependence, posing urgent issues regarding the formulation of a sustainable production covering such aspects as natural capital or food security [96,97].

However, recently researchers have been calling for a reconsideration of depicting soy production as the capitalist, large-scale farming narrative leaving no place for the recognition of small-scale production. While soybean’s sustainability discourse focuses mostly on these large actors embedded in the transnational agri-food commodity chain, research has to reconsider different farming styles in order to recognize sustainable solutions outside of this large-scale–small-scale dichotomy [94,98]. In this framework soy can rather be connected to the technological advancement and modernization of agriculture in contrast to quinoa’s heritage rooting in the traditional cultivating territories and their people. Furthermore, it can serve as a cautionary regarding the impact evaluation of turning quinoa into a global commodity and scale as a key question in evaluating sustainability potentials. 

The case of aҫaí berries can provide us a basis for a comparative case study, rather than a cautionary tale. The production and market for aҫaí berry is similar to the ones of quinoa from many aspects. Firstly, it is considered a superfood as well, due to its high nutritional value and its market has great potentials with increasing demand. Aҫaí is traditionally grown in Brazilian Amazon (*Euterpe Oleraceae Mart*, from the eastern part and *Euterpe precatoria Mart*, in the central and western part [99]. Similar to quinoa, it constitutes not only a key element of the local’s diet but it also provides a source of income. Following the growing demand for açaí and market expansion, the internationalization process has begun with businesses appearing outside of Brazil, such as Sambazon from California [100]. These factors certainly raise the same questions regarding socioeconomic and environmental impacts of increased demand, as in the case of quinoa. 

Income dependence due to seasonality and price fluctuations, new management practices, and the future of supply-demand balance can put producing communities in vulnerable position [101,102]. The introduction of monoculture and “modern production models” can bear further negative impacts to local communities [101] (p. 944). On the other hand, the extraction of aҫaí, as a non-timber forest product (NTFP) has great opportunities in terms of contributing to local livelihoods or reducing deforestation, as the growing prices can provide a good income for extractors. Lopes et al. analyzed Acre region in terms of açaí production potentials and concluded the region could provide a sufficient place for sustainable production, despite the fact that NTFPs are still diminishing in terms of income potential next to cattle ranching [99]. Concluding this parallel, in terms of opportunities and threats to sustainable aҫaí production, similar dynamics can be noted as in the case of quinoa, such as income dependence, dietary changes in traditional producing communities, depletion of natural resources and adapting new management and production techniques. In addition to identifying these potential uses and market value of quinoa, the growing demand driving pressuring market dynamics points out the need to identify the necessary conditions for enhancing a sustainable production of quinoa both from ecological and societal aspects. The potentialities of natural resources are given, but balancing the market demand and supply is inevitable to avoid further destructive social and ecological impacts affecting producing communities. For understanding this balance, a multidisciplinary approach is needed in order to map out the interconnections between the crop embedded in the landscape and the communities themselves. Consequently, this overview is calling for the reconsideration of the crop as a sustainable solution in order to broaden the conceptual framework with putting further focus on socio-economic and environmental impacts in traditional producing countries. 

### 7.2. New Product Development and Corporate Responsibilty

What can traders and processors do better for the future of quinoa’s producers, for its environmental and social impact and for consumers? For some scholars the answer lies in functional food development (FFPD) as the future for new product development (NPD) [48,51]. Instead of being market-oriented, FFPD tends to be more product-oriented and exploit technical opportunities to develop new markets, hence consumers also have specific information [103]. Moreover, NPD generate knowledge using trial and error method with prevailing knowledge whereas the successor tends to explore and analyze new knowledge in order to outperform others [48,104]. Additionally, FFPD resource base is an open-source, which is open to both the development of market, production line, technical skills and innovative ideas from external partners [105,106]. This is not the case for NPD, as it focuses on internal database with limited external partners from whom data is collected, such as material suppliers [107]. FFPD commercial strategy involves multi-stakeholders who interact, and the firm focuses on maintaining trust and relationship, which was weakened [108]. Competition is enhanced with further inclusion of external stakeholders such as medical and research institutions, input suppliers, pharmaceuticals, research organizations [109]. FFPD firms focus on radical innovation and not incremental innovations as their goals are not to minimize costs and focus on long term goals unlike the current NPD. It is evident from the environmental and socio-economic issues that innovative functional foods are in need of a shift in paradigm towards radical food innovations. A collaboration of resources is not only cost and time effective but also puts firms in a position to decide regarding an optimal outsource plan [51]. 

It is also essential to find out the corporate social responsibility (CSR) of profit-oriented firms and to review whether they are misusing quinoa for purely private profits [51]. Walsh-Dilley has argued that ‘cooperation’ amongst other members of the community alongside ‘reciprocity’ would be the way towards a moral economy [72]. Additionally, Sayer wrote: “I use the term moral economy both to describe a system of livelihood institutions and practices and as a mode of inquiry that is oriented to the ‘study of how economic activities of all kinds are influenced and structured by moral dispositions and norms, and how in turn those norms may be compromised, overridden or reinforced by economic pressures” [72] (page 78). In highland Bolivia, marketization and upgrading would not infect the farmers. Rather it would consolidate and may further improve the system, as this mixture of two systems would lead to a hybrid economy i.e., capitalism along with reciprocity [72]. Pursuing economics from a moral aspect and market logic delivers a strong toolkit that involves the Andean people to cope with the unevenness of completely getting involved in global capitalism [72,110]. Lastly, technological advances cannot flourish limitlessly, as quinoa is a skilled labor-intensive crop. It is necessary to understand the balance of market power between the Global North and South alongside the understanding whether an economic system can hold morality as a virtue within its actions as an open market economy [48,111]. Researchers have feared that social cooperation and reciprocity which happen to be strong features of farming in the Andes are threatened, as they have come into contact with capitalism and neoliberal individualism [72]. Others have researched that increasing prices of 1% increases the producer’s welfare by 0.07%. Nevertheless, welfare increase only occurs for the duration of booms in quinoa price in the international market. A rise in prices can have two welfare benefits for quinoa producers, i.e., higher welfare and reduced variability of household welfare [79]. 

## 8. Conclusions

Quinoa is emerging as a quality source of protein, fiber mineral, and bioactives. It has been exploited in the development of gluten-free and nutrient-enriched novel food products. In addition, studies have been conducted for its nonconventional applications such as its use as a nutraceutical, the use of quinoa starch-based edible films and/or packaging and stabilizers, and quinoa protein-based packaging films, among others.

As seen in the presented tables, cultivars perform differently when grown in different locations. Thus, the relevance of the improving varieties is key towards a sustainable growth and further expansion of quinoa worldwide. Breeding programs conducted in the last decades have led to the development of various cultivars adapted to different agroecological conditions, aiming maximized yields. However, it is still questionable if such breeding objectives have considered seed nutritional quality. It is therefore important that future breeding programs include participatory approaches to understand and define breeding objectives that promote the adaptation of cultivars to marginalized conditions; thus, encompassing climatic challenges that smallholders farmers will face in the future, promoting nutritional security for those in threat. In addition, although numerous studies have been published in the last years describing the effects of abiotic stress in quinoa plants, a transdisciplinary-perspective analysis of how the cultivars respond to a range of environments-management are still yet to be explored. Such responses may flourish understanding by sensitive genotype-environment interactions. Investigations of how such interactions alter the nutritional composition of plant parts for particular cultivars and selected genetic traits response to abiotic stresses is important to be scrutinized by the scholarship.

Moreover, a crescent awareness in using minor biochemical components present in quinoa poses an interesting scenario to the development of alternative ingredients/raw materials for industrial use. The promotion of new technologies and the use of such chemicals can foster the value chain of quinoa and producing regions. Consequently, side stream processing of waste from quinoa production in agriculture, topical applications, feedstock, and bioenergy has great potential to be expanded upon in the future. This can provide a valuable side stream of revenue from the quinoa production and processing for the quinoa producers, while at the same time offering health benefits to consumers. This comes with a cost that neither the industry nor society can afford any longer. The consequences that come from the various market decisions will impact sustainability protocols, which will have social, economic and environmental consequences. If a switch in our diets is needed, if we take seriously the demand for healthy, sustainable, ethical food products and goods as a demand for pursuing sustainability, then we have to learn from the paths we have walked on so far, and act accordingly.

Lastly the sustainability discourse, which currently focuses on mostly on mitigating the negative impacts on the producing countries, should incorporate the objective to create fair access to the global and local market. This takes into consideration the question of ownership regarding quinoa from Andean nations as a strategic approach towards adapting sustainable production. For adjusting this strategic path, a well-defined scientific research agenda, responsible and participatory cooperation, strong political will from national governments and international organizations, and consumer awareness is needed.

## Figures and Tables

**Table 1:** Proximate composition of quinoa seeds cultivated in different regions.

Growing Year	Country	Location	Cultivar	Observation	Carbohydrate	Protein	Fat	Fiber	Ash	Reference
					(values in % or g 100 g^−1^ Seeds DM)					
1998	Bolivia		Real		63.7	12.9	6.5	13.9 *	3.0	[12]
2006–07	Italy	Vitulazio	KVLQ520Y	early sow	55.6	16.2	7.8	16.1 *	4.3	[13]
				late sow	54.8	16.2	7.7	16.9 *	4.1	
			Regalona Baer		52.8	16.8	7.9	18.6 *	4.0	
2006–09	Argentina	Salta and Jujuy	mean value of 21 data entries		51.4	16.8	5.9	12.1 *	4.7	[9]
2010	Chile	North	Ancovinto		68.1	13.0	6.2	1.5	3.4	[14]
			Cancosa		65.8	13.6	6.0	1.8	3.5	
		Center	Cáhuil		64.2	11.1	7.1	1.2	3.2	
			Faro		63.8	11.4	6.7	1.6	3.5	
		South	Regalona		59.4	14.4	6.4	1.8	3.7	
			Villarrica		56.5	16.2	5.6	2.9	3.7	
2010	Peru	Cusco	ND			13.2	6.5	4.2	2.3	[15]
			ND			13.5	6.3	7.0	2.3	
		Puno	03-21-0093			11.8	-	-	2.8	
			03-21-1181			13.5	4.0	2.9	3.1	
			Coito			14.7	5.3	1.8	2.8	
			Huaripongo			13.2	6.1	2.5	2.9	
			INIA-415 Pasankalla			12.7	6.9	2.2	2.5	
			Roja de Coporaque			11.5	5.2	2.3	2.9	
			Salcedo			13.2	5.3	1.8	2.4	
			Witulla			12.3	5.3	2.6	2.6	
2011			La Molina 89			13.6	6.0	3.0	4.8	
		Puno	Blanca de Juli			12.4	4.9	1.8	3.0	
			Kcancolla			13.5	5.1	2.7	3.1	
			Sajama			12.7	4.1	1.7	2.7	
2010	Italy	Vitulazio	Titicaca, Q100	100% irrigation	49.0	14.6	5.1	17.6 *	3.4	
			Titicaca, Q25	25% irrigation	49.9	14.4	5.2	14.6 *	3.3	
			Titicaca, Q50	50% irrigation	51.9	14.7	5.1	16.9 *	3.5	
			Titicaca, Q100S	same irrigation as above but with saline water	49.7	13.3	5.2	19.5 *	3.7	
			Titicaca, Q25S		48.6	13.3	4.7	18.7 *	3.5	
			Titicaca, Q50S		49.0	14.0	5.2	17.5 *	3.3	
2013	Peru	Mantavaro valley	Ayni			14.8	4.7			[16]
2015	USA			USDA database	57.2	14.1	6.1		2.4	[17]
				Various primary sources ^†^	59.9	13.1	5.7	3.3	3.3	
2015	Germany	Stuttgart	Zeno			12.0	5.5 ^‡^			[18]
			Jessie			16.1	7.3 ^‡^			
			Puno			13.0	6.5 ^‡^			
			Titicaca			13.4	7.5 ^‡^			
2016	Germany	Stuttgart	Zeno			12.0	5.5 ^‡^			
			Jessie			13.1	7.3 ^‡^			
			Puno			13.0	6.5 ^‡^			
			Titicaca			12.3	7.5 ^‡^			
2016	Chile	Río Hurtado	Regalona			15.2		3.1		[11]
			Salcedo			18.1		3.3		
			Titicaca			16.4		3.6		
2016	Spain	El Pobo	Regalona			17.8		3.0		
			Salcedo			15.7		3.2		
			Titicaca			15.3		3.5		
2016	Peru	Arequipa	Salcedo			14.6		3.3		

* values for fiber are reported as total dietary fiber. ^†^ n= 34 for carbohydrate, 37 for protein, 37 for fat, 23 for fiber, and 37 for ash. ^‡^ mean values for two growing years.

**Table 2:** Amino acid composition of quinoa seeds (g 100 g^−1^ crude protein).

			Essential									Semi-Essential					Non-Essential							
Year	Country	Variety	Ile	Leu	Lys	Met	Phe	Thr	Trp	Val	His	Cys	Tyr	Gly	Arg	Pro	Ser	Asp	Glu	Ala	Asn	Hyp	Glu	Reference
2010	Chile	Ancovint	3.8	6.8	4.2	1.4	4.1	3.5	-	4.9	2.7	-	2.8	4.4	10.7	7.1	4.2	6.6	-	4.6	-	-	10.9	[14]
		Cancosa	3.4	6.5	4.1	1.5	3.9	3.2	-	4.6	2.8	-	2.8	4.5	10.9	7.7	4.1	6.9	-	4.2	-	-	10.8	
		Cáhuil	2.9	6.4	4.1	1.7	3.9	3.3	-	4.7	2.7	-	3.1	5.3	10.9	9.4	4.1	5.5	-	4.5	-	-	10.7	
		Faro	3.4	7.0	4.4	1.7	4.2	3.6	-	4.9	3.1	-	3.3	5.4	12.0	9.0	4.4	7.0	-	4.7	-	-	11.0	
		Regalona	3.0	6.6	4.3	1.7	4.0	3.3	-	4.3	3.0	-	2.9	5.4	11.9	7.4	4.3	6.5	-	4.2	-	-	11.5	
		Villarrica	3.1	7.2	4.8	1.9	4.5	3.4	-	4.4	3.5	-	3.1	6.1	11.9	6.7	4.8	6.7	-	4.5	-	-	11.4	
2015	USDA		3.6	5.9	5.4	2.2	-	3.0	1.2	4.2	2.9	1.4	-	-	-	-	-	-	-	-	-	-	-	[17]
2015	Germany	Zeno	2.0	3.7	2.8	1.1	2.2	2.1	1.0	4.2	1.3	1.0	1.6	3.0	3.8	2.3	2.8	5.2	6.9	2.8	-	-	-	[18]
		Jessie	2.4	4.3	3.5	1.4	2.7	2.6	0.9	4.4	1.8	1.2	2.0	3.8	5.2	2.7	3.3	6.0	8.5	3.3	-	-	-	
		Puno	3.2	5.4	4.0	1.5	3.6	3.3	1.0	4.0	1.9	1.3	2.3	4.7	5.2	3.1	3.8	7.1	11.8	3.9	-	-	-	
		Titicaca	2.7	4.8	3.7	1.4	3.0	2.8	0.9	4.9	1.9	1.2	2.0	4.1	5.2	3.2	3.3	5.9	8.3	3.3	-	-	-	
2016	Germany	Zeno	2.5	4.5	4.0	1.4	2.8	2.6	0.9	4.4	1.9	1.1	1.9	3.7	5.6	2.9	2.8	5.2	6.9	2.8	-	-	-	
		Jessie	2.8	5.3	4.9	1.8	3.2	3.2	1.0	5.7	2.3	1.4	2.3	4.6	6.6	3.2	3.3	6.0	8.5	3.3	-	-	-	
		Puno	3.2	5.6	5.0	1.8	3.5	3.2	1.1	3.8	2.5	1.5	2.4	5.0	7.5	3.3	3.8	7.1	11.8	3.9	-	-	-	
		Titicaca	2.6	4.6	4.2	1.6	2.8	2.7	1.0	4.9	2.0	1.3	2.0	4.1	6.0	3.0	3.3	5.9	8.3	3.3	-	-	-	

**Table 3:** Essential amino acid profile of quinoa and other grains, compared to the FAO recommended amino acid scoring pattern for older children (3 to 10 years old), adolescents, and adults [20,21,22].

Amino Acids	FAO	Quinoa	Maize	Rice	Wheat
Isoleucine	3.0	4.9	4.0	4.1	4.2
Leucine	6.1	6.6	12.5	8.2	6.8
Lysine	4.8	6.0	2.9	3.8	2.6
Methionine	2.3	5.3	4.0	3.6	3.7
Phenylalanine	4.1	6.9	8.6	10.5	8.2
Threonine	2.5	3.7	3.8	3.8	2.8
Tryptophan	0.7	0.9	0.7	1.1	1.2
Valine	4.0	4.5	5.0	6.1	4.4

**Table 4:** Most relevant fatty acids content of quinoa seeds.

	Fatty Acid Profile								
	Saturated			Unsaturated					
Variety	C16:0	C18:0	C23:0	C18:1 n-9	C18:1 n-7	C18:2	C18:3-α	C18:3-γ	Reference
21 accessions				25.40		50.40	6.6		[9] *
Ancovinto	7.87	0.75	4.44	27.87		45.17	8.30	0.51	[14] ^†^
Cancosa	8.14	0.70	3.49	26.91		46.57	8.27	0.50	
Cáhuil	8.32	0.63	4.30	23.45		52.90	5.45	0.49	
Faro	8.19	0.67	4.88	22.25		53.89	4.64	0.48	
Regalona	8.56	0.61	6.81	18.68		54.18	5.35	0.43	
Villarrica	8.97	0.54	3.79	20.77		53.36	5.88	0.34	
Ayni	96.00	26.00		239.00	8.00	488.00	49.00		[16] ^‡^
Zeno	6.96	0.45		13.14	0.92	40.67		4.55	[18] ^†^
Jessie	8.56	0.65		16.55	1.04	45.68		4.98	
Puno	8.48	0.71		14.41	1.07	40.39		4.59	
Titicaca	6.97	0.45		13.08	0.79	33.07		3.29	

* Reported values are average for 21 accessions (from Northwest Argentina) in g 100 g^−1^ of total fatty acids. ^†^ Reported values in g 100 g^−1^ fat. ^‡^ Reported values in g kg^−1^ of total fatty acids.

**Table 5:** Mineral content of quinoa and other grains [21,22].

Mineral (mg 100 g^−1^ Seeds DM)	Quinoa	Maize	Rice	Wheat
Calcium	148.7	17.1	6.9	50.3
Iron	13.2	2.1	0.7	3.8
Magnesium	249.6	137.1	73.5	169.4
Phosphorus	383.7	292.6	137.8	467.7
Potassium	926.7	377.1	118.3	578.3
Zinc	4.4	2.9	0.6	4.7

**Table 6:** The mineral content of quinoa seeds of different varieties.

Year	Country	Location	Variety	Ca	Fe	Mg	P	K	Na	Zn	Cu	Mn	Reference
				mg kg^−1^ Seed DM									
2003	Peru		Huancayo	940.0	168.0	2700.0	1400.0		115.0	48.0	37.0		[23]
2004				863.0	150.0	5020.0	4110.0	7320.0		40.0			[27]
2006				1274.0	20.0		3869.0	6967.0		48.0			[24]
2009				565.0	14.0	1760.0	4689.0	11,930.0		28.0			[28]
2016	Chile	Río Hurtado	Regalona	1265.5	91.0	2278.5	3437.9	13,856.5	12.1	40.9			[11]
			Salcedo	1360.2	83.3	2238.1	3246.1	10,006.3	11.4	42.7			
			Titicaca	619.0	82.5	1814.0	2846.4	10,250.3	5.2	40.8			
	Spain	El Pobo	Regalona	729.0	55.4	1962.9	4232.9	11,440.3	3117.0	25.4			
			Salcedo	934.5	66.8	1741.2	3155.8	8866.9	16.7	25.3			
			Titicaca	888.4	69.3	1863.9	3915.4	14,678.5	16.7	25.1			
	Peru	Arequipa	Salcedo	514.0	62.8	1924.1	3934.6	9648.7	5147.0	33.0			
2015	Various			870.0	94.7	3620.0	4060.0	9070.0	200.0	21.5	78.4		[17]
	Bolivia			1130.0	50.2		2510.0						
	Peru			630.0	84.7		2730.0			37.3			
	USA			540.0	52.7	2270.0	5270.0	6490.0	60.0	35.7	6.8		

**Table 7:** Saponin content of quinoa seeds of different varieties.

Year	Country	Location	Variety	Saponin	Reference
				g 100 g^−1^ Seed DM	
2006–07	Italy	Vitulazio	Regalona Baer	3.3	[13]
2016	Chile	Río Hurtado	Regalona	1.3	[11]
			Salcedo	1.0	
			Titicaca	1.2	
	Spain	El Pobo	Regalona	1.4	
			Salcedo	0.9	
			Titicaca	1.3	
	Peru	Arequipa	Salcedo	0.8	
2015	Germany	Stuttgart	Zeno	2.7	[18] *
			Jessie	0.7	
			Puno	2.6	
			Titicaca	2.6	
2016	Germany	Stuttgart	Zeno	2.8	
			Jessie	0.0	
			Puno	2.9	
			Titicaca	3.4	
	Argentina		Sajama	0.8	[31]
			N.R.	2.9	
	Bolivia		Real	2.6	
	Brazil		BRS-Piabiru	3.3	
	Denmark		Olav	1.8	
			Q52	6.1	

* mean value of two years in mg g^−1.^

**Table 8:** The vitamin content of quinoa seeds compared to other grains (mg 100 g^−1^ DM) [21,22].

Vitamin	Quinoa	Maize	Rice	Wheat
Thiamine	0.2–0.4	0.42	0.06	0.45–0.49
Riboflavin	0.2–0.3	0.1	0.06	0.17
Folic Acid	0.08	0.03	0.02	0.08
Niacin	0.5–0.7	1.8	1.9	5.5

**Table 9:** Summary of cause of inefficiencies in traditional post-harvest processing of quinoa and solutions offered [33].

Cause of Inefficiencies	Solutions
Adaptation of technologies developed for processing other grains in an inadequate manner for processing quinoa (e.g., scarification)	An efficient system of dry cleaning was designed, constructed and implemented, that made use of the inherent abrasive properties of quinoa for scarification
Use of washing systems with a wide and variable range of residence time, leading to product variability (not all grains are washed for the correct amount of time)	A washer was designed with the objective of accomplishing the simulation of a laminar trajectory of the grain, using a turbulent flow, thus, creating a homogenous process. In addition, the residence time was also reduced
Use of drying systems with insufficient air flows, thus allowing part of the product to re-humidify	Development of a more efficient drying system, making use of a turbine
Use of technologies that did not focus on or allow for the recovery of sub-products of high commercial value (e.g., saponins)	A saponin recovery system was installed to recover this important sub-product which has high economic value in the market
Use of technologies that operated in small batches, instead of a continuous process	The new technology operates in a continuous manner and uses fewer operators
Excessive and unnecessary number of unit operations in the process	The installed technology is more efficient in the drying and washing steps and therefore no addiction cleaning operations are needed

**Table 10:** Installed processing capacity of quinoa in Bolivia [33].

Company	Installed Capacity Before Technological Innovations (Tons/Year)	Installed Capacity After Technological Innovations (Tons/Year)	Processing Capacity Increase (%)
AVSA	240	1900	792
ANAPQUI	920	2800	304
CECAOT	440	2800	636
QUINOABOL	600	2800	467
IRUPANA	600	2800	467
CEREALES INDINA	50	2800	5600

**Table 11:** Traditional quinoa food products adapted from Bojanic (2011) [6].

Product	Description
Quinoa Soup	Cooked quinoa with meat, tubers and vegetables
Lawa	A porridge like dish with raw flour, water with lime and animal fat
P’esque	Quinoa grain cooked without salt, served with milk or grated cheese
Kispiña	Steamed buns
Tacti o tactacho	Fried buns, like a doughnut, from flour and llama fat
Mucuna	Steam cooked balls from quinoa flour, filled with seasoning
Phiri	Roasted and slightly dampened quinoa flour
Phisara	Lightly roasted and cooked quinoa grain
Q’usa	Quinoa chicha, a macerated cold drink
El Ullphu, Ullphi	Cold drink prepared with roasted quinoa flour
Kaswira de quinua	Flattened bread fried in oil with lime and white quinoa
Kaswira de ajara	Flattened bread fried in oil with lime and black quinoa or ajara
K’api kispiña	Steamed bun with quinoa ground
Turucha quispiña o Polonca	Large steamed breads made with katahui and quinoa lightly ground (chama)
Mululsito quispiña	Steamed bread, made with katahui and quinoa flour
Quichi quispiña	Steamed and fried bread, made with katahui and quinoa flour
Juchcha	Andean soup based on ground quinoa and katahui
Chiwa	Young quinoa leaves are used as a vegetable in salads and soups
